# Polyphenol-Related Gut Metabotype Signatures Linked to Quality of Life in Postmenopausal Women: A Randomized, Placebo-Controlled Crossover Trial

**DOI:** 10.3390/nu17223572

**Published:** 2025-11-15

**Authors:** María P. Jarrín-Orozco, María Romo-Vaquero, Concepción Carrascosa, Miriam Pertegal, José Berná, Julio Puigcerver, Adrián Saura-Sanmartín, Isabel Espinosa-Salinas, María García-Nicolás, María Á. Ávila-Gálvez, Juan C. Espín

**Affiliations:** 1 Laboratory of Food & Health, Research Group on Quality, Safety, and Bioactivity of Plant Foods, CEBAS-CSIC, Campus de Espinardo, 30100 Murcia, Spain; mpjarrin@cebas.csic.es (M.P.J.-O.); mrvaquero@cebas.csic.es (M.R.-V.); mgnicolas@cebas.csic.es (M.G.-N.); mavila@cebas.csic.es (M.Á.Á.-G.); 2 Department of Obstetrics and Gynecology, Virgen de La Arrixaca University Hospital, El Palmar, 30120 Murcia, Spain; conchicarrascosa@yahoo.es (C.C.); miriam.pertegal@gmail.com (M.P.); 3 Department of Organic Chemistry, Faculty of Chemistry, University of Murcia, 30100 Murcia, Spain; ppberna@um.es (J.B.); julio.puigcervera@um.es (J.P.); adrian.saura@um.es (A.S.-S.); 4 IMDEA Food, CEI UAM+CSIC, Carretera de Cantoblanco, 8. 28049 Madrid, Spain; mariaisabel.espinosa@imdea.org

**Keywords:** urolithin, equol, lunularin, pomegranate, isoflavone, resveratrol, personalized nutrition, menopause, hot flashes, Cervantes scale

## Abstract

**Background/Objectives**: Interindividual variability in polyphenol metabolism may help explain the inconsistent effects of polyphenol intake on health outcomes. This study compared, for the first time, (i) polyphenol-related gut microbiota metabotypes (urolithins: UM0, UMA, UMB; equol: EP, ENP; lunularin: LP, LNP) and their clusters (MCs) in non-medicated premenopausal (Pre-M) and postmenopausal (Post-M) women and (ii) the impact of an 8-week intake of a polyphenol-rich plant extract mixture (PPs) on the quality of life (QoL) of Post-M. **Methods**: Polyphenol metabotypes were determined in urine via UPLC-QTOF-MS after a 3-day intake of PPs containing resveratrol, pomegranate (ellagitannins and ellagic acid), and red clover (isoflavones) in Pre-M (*n* = 120) and Post-M (*n* = 90) women. QoL was assessed with the short-form Cervantes Scale in a randomized, placebo-controlled crossover trial (8-week PPs vs. placebo), completed by 78 Post-M participants. **Results**: At baseline, Pre-M and Post-M women showed only minor differences in metabotype and MC distributions linked to menopausal status. MC3 (UMA+EP+LP) predominated in Pre-M, while MC7 (UMA+EP+LNP) was most frequent in Post-M. PPs intake in Post-M women led to modest shifts in metabotype and MC distributions toward Pre-M patterns. Quantitative metabolite production was comparable between groups, except for equol, which showed a median 2.8-fold increase after PPs intake in EP Post-M women. Clinically meaningful improvements (score reduction ≥ 6.7 points) in QoL were observed in the Psychic domain in EP women (28%, *p* = 0.039) and in the Menopause and Health domain, specifically in EP (24.1%, *p* = 0.004), MC3 (22.5%, *p* = 0.043), and MC4 (UMB+EP+LP; 41.3%, *p* = 0.022), were mainly driven by a reduction in hot flashes (*p* = 0.001). **Conclusions**: These findings support metabotyping as a tool to guide targeted dietary strategies and enhance QoL through precision health in Post-M women.

## 1. Introduction

Menopause, the permanent cessation of menstruation due to declining estrogen and progesterone levels, typically occurs between ages 50 and 52 and is associated with physical and psychological symptoms that can impair health-related quality of life (QoL) [1,2,3].

The Cervantes scale was developed to assess menopausal effects on the QoL of postmenopausal (Post-M) women [4]. Its short form (C-SF) includes four dimensions (menopause and health, psychology, sexuality, and couple relationship) and captures the same constructs with simplified scoring [5,6].

Phenolic compounds, hereafter referred to as polyphenols, are bioactive dietary compounds with potential benefits against chronic diseases through mechanisms such as lipid metabolism, inflammation, epigenetic regulation, and autophagy [7,8,9,10]. However, the majority of studies have failed to demonstrate consistent associations between polyphenol intake and health outcomes, prompting the European Food Safety Authority (EFSA) to reject most health-related claims, primarily due to the high interindividual variability observed [11].

This controversy has also been observed in studies related to menopause and polyphenols. Numerous meta-analyses based on clinical studies support the benefits of isoflavones for women’s health after menopause, particularly in the prevention of cardiometabolic diseases and the management of postmenopausal symptoms [12,13,14]. However, several meta-analyses of clinical studies have highlighted controversial outcomes, either by failing to confirm these effects consistently or by emphasizing the heterogeneous results among individuals [15,16,17]. The effects of other polyphenols, including resveratrol (RSV), have been less extensively investigated in postmenopausal women, but findings remain inconclusive [18,19,20]. Overall, this controversy may reflect interindividual variability in polyphenol metabolism, resulting in metabolites with varying biological activities.

The unequal response of individuals to polyphenol consumption has led to the concept of polyphenol-related gut microbiota metabotypes, which categorize individuals based on their ability to produce specific gut microbial metabolites with distinct bioactivities [9,21,22]. Three metabotypes have been described to date: (i) equol producers (EPs) versus non-producers (ENPs), derived from the metabolism of isoflavones, mainly daidzein [23]; (ii) urolithin metabotypes, resulting from ellagitannin and ellagic acid metabolism (found in walnuts, pomegranate, berries, and tea), further classified as metabotype A (urolithin A producers, UMA), metabotype B (producers of urolithin A, isourolithin A, and urolithin B, UMB), and metabotype 0 (non-producers, UM0) [24]; and (iii) lunularin producers (LPs) versus non-producers (LNPs), derived from resveratrol metabolism [25]. Adding to this complexity, up to 12 possible combinations of these metabotypes, termed metabotype clusters (MCs), can occur when consuming isoflavones, resveratrol, and ellagic acid, as a result of the combination of the individual metabotypes (e.g., UMA+EP+LP, UMB+ENP+LP, etc.) [22]. This raises the hypothesis that specific metabotypes or their combinations may predict individual responses to polyphenol intake, particularly in relation to quality of life in postmenopausal women [9].

However, evidence linking polyphenol-related metabotypes to health outcomes through randomized clinical trials remains limited. Some associations have been reported in relation to the differential production of urolithins [26,27], with urolithin A (Uro-A) considered the most bioactive metabolite [28,29], and in the metabolism of isoflavones, where equol is recognized as the main bioactive metabolite [9,23,30]. In contrast, no controlled trials or association studies have yet examined the relationship between resveratrol-related metabotypes (LP and LNP) and human health [9]. Furthermore, the distribution of urolithin metabotypes can vary with age or chronic medication [9,31,32], and equol production is influenced by the body mass index (BMI) [23,33].

To address this gap, we hypothesized that menopausal status, independently of medication, may influence the capacity to produce urolithins, equol, and lunularin, and that the resulting metabotypes and their combinations (MCs) may modulate the effects of polyphenol intake on QoL. Accordingly, we (i) compared these metabotypes and MCs between premenopausal (Pre-M) and postmenopausal (Post-M) women and (ii) evaluated, in a randomized, double-blind, placebo-controlled crossover trial, whether an 8-week intake of a mixture of plant extracts rich in isoflavones, ellagic acid, and RSV exerts metabotype- or MC-dependent effects on the QoL of Post-M women, and whether the intervention alters metabotype and MC distributions.

## 2. Materials and Methods

### 2.1. Reagents

High-performance liquid chromatography (HPLC)-grade acetonitrile, dimethyl sulfoxide (DMSO), formic acid, and methanol were obtained from JT Baker (Deventer, The Netherlands). *trans*-Resveratrol (resveratrol, RSV), ellagic acid (EA), daidzein, genistein, formononetin, biochanin A, equol, and hesperetin were purchased from Sigma-Aldrich (St. Louis, MO, USA). Equol 7-O-glucuronide and O-demethylangolensin (ODMA) were purchased from LGC Standards (Barcelona, Spain). RSV 4′-O-sulfate, RSV 3-O-glucuronide, dihydroresveratrol 3-O-glucuronide, RSV 3-O-sulfate, Urolithin A (Uro-A), B (Uro-B), Isourolithin A (IsoUro-A), Uro-A 3-O-glucuronide, Uro-A 8-O-glucuronide, Uro-B glucuronide, Uro-A sulfate, Uro-B sulfate, IsoUro-A 3-O-glucuronide, IsoUro-A 9-O-glucuronide, lunularin (LUNU), 4-hydroxydibenzyl (4HDB), LUNU glucuronide, LUNU sulfate, 4HDB glucuronide, and 4HDB sulfate were obtained as described elsewhere [25,34,35]. All the reagents and metabolites were 97% pure or higher. Ultrapure Millipore water (Darmstadt, Germany) was used throughout the study.

### 2.2. Polyphenol-Rich Supplement (PPs)

The polyphenol-rich supplement (PPs) consisted of a mixture of RSV and commercially available pomegranate and red clover extracts. The components were mixed and encapsulated in hard gelatin capsules by Laboratorios Admira S.L. (Alcantarilla, Murcia, Spain). The capsules were manufactured, tested, and checked in accordance with the European Union’s Good Manufacturing Practices requirements. Each capsule (700 mg) contained a mixture of 50 mg *trans*-RSV (98% purity) from Polygonum cuspidatum, 320 mg punicalagin-rich pomegranate extract (20% punicalagin), 80 mg EA-rich pomegranate extract (40% EA), and 250 mg red clover extract (20% isoflavones). The PPs mixture was analyzed by High-Performance Liquid Chromatography coupled to Electrospray Ionization-Ion Trap Tandem Mass Spectrometry (HPLC-ESI-IT-MS/MS), as previously described [36], and its detailed composition is shown in Table 1. The placebo capsules (Pla) contained microcrystalline cellulose (700 mg) and were indistinguishable from those containing the extract mixture.

### 2.3. Study Design and Participants

The study protocol was approved by the Bioethics Committee of the Spanish National Research Council (CSIC) (Madrid, Spain) (reference 249/2023), IMDEA-Food (Madrid) (reference PI-065), and the Virgen de La Arrixaca University Hospital (Murcia, Spain) (reference 2023-4-9-HCUVA) within the PolyPAUSE Project (PID2022-136419OB-I00; MICIU, Spain). The trial adhered to the principles of good clinical practice outlined in the Declaration of Helsinki of 1975 and its subsequent amendments, and it was registered at clinicaltrials.gov (NCT07182370). Participants were enrolled from September 2023 to January 2025.

The trial consisted of two studies: Study 1 involved women of reproductive age (premenopausal; Pre-M) with a 3-day follow-up for metabotype stratification, and Study 2 included Post-M women with an initial 3-day follow-up for metabotype stratification and an 8-week follow-up to assess the impact of PPs on QoL.

***Study 1.*** Healthy Pre-M women (aged 30−48 years) were recruited at the IMDEA Food Institute (Madrid) and CEBAS-CSIC (Murcia, Spain). Exclusion criteria were as follows: intake of antibiotics within the month before the study; use of chronic medication or any other medication, even sporadically, within one month before the start of the study; BMI < 18 kg/m^2^; swallowing difficulties; pregnancy or lactation; smoking; diagnosed chronic illness; previous gastrointestinal surgery; habitual alcohol consumption > 15 g/day; vegetarian diet; allergy, intolerance, or regular consumption of red clover extract, resveratrol (grape, wine), soy, or pomegranate; or current participation in a weight-loss regimen. The study was fully explained to the volunteers, who provided written informed consent before participation. Physical activity levels were assessed using the validated International Physical Activity Questionnaire (IPAQ) [37]. Adherence to the Mediterranean diet was recorded using the validated PREDIMED questionnaire [38].

Pre-M participants consumed three capsules per day in the evening, containing the polyphenol-rich extract mixture (PPs), for three consecutive days. Participants were instructed to take the capsules in the evening to standardize for potential circadian effects and, in particular, to maximize the likelihood of detecting polyphenol-derived metabolites in the first morning urine sample after fasting. The daily intake of PPs (3 capsules) provided 133.2 ± 10.1 mg of RSV, 312.0 ± 30.9 mg of ellagitannins (including punicalin, α- and β-punicalagins) and free ellagic acid, as well as 166.3 ± 27.3 mg of isoflavones, including daidzein, genistein, formononetin, and biochanin A. At the end of the study, the participants provided the questionnaires and a urine sample. The aim was to stratify this group of women according to their polyphenol metabotype and corresponding clusters (MCs) [22].

***Study 2.*** Healthy postmenopausal women (Post-M) were recruited at IMDEA Food, CEBAS-CSIC, and the Virgen de La Arrixaca University Hospital (Murcia). Inclusion criteria were the following: women aged 45–59 years, with menopause (defined as ≥1 year without menstruation), presenting at least one climacteric symptom (hot flashes, sweating episodes, low mood, irritability, altered libido, insomnia, and/or joint or muscle pain), and with a BMI > 18 kg/m^2^. Exclusion criteria were as follows: chronic-degenerative or psychiatric conditions; medical contraindications to nutritional supplements; chronic medication use (e.g., cholesterol, blood pressure, hypertension, anxiety, insomnia, or others) or any other medication, even sporadically, within one month before the start of the study; antibiotic use within the month before enrollment; hormone replacement therapy; history of major gastrointestinal surgery; swallowing difficulties; BMI < 18 kg/m^2^; current participation in a weight-loss regimen; allergy or intolerance to red clover extract, resveratrol (grape, wine), soy, or pomegranate; smoking; habitual alcohol consumption > 15 g/day; vegetarian diet; or regular intake of supplements containing resveratrol, pomegranate, or isoflavones. Participants received a comprehensive explanation of the study and provided written informed consent before enrollment. Physical activity level and adherence to the Mediterranean diet were also recorded as described above. The primary outcome of the trial was the distribution of metabotypes and their combinations (i.e., metabotype clusters, MCs) in Post-Mwomen. A sample size of 90 participants was considered based on prior studies and the logistical difficulty of recruiting medication-free volunteers [22]. The impact of PPs intake on QoL in Post-M women was defined as a secondary outcome. With 80% power, 95% confidence, and an anticipated 15% dropout rate, a minimum of 40 completers was considered necessary to provide an initial evaluation of QoL changes, as reported elsewhere [39].

Three days before and during the intervention, including the washout period, Post-M participants consumed a low-polyphenol diet supervised by a nutritionist and based on pasta, grilled meat or fish, low-fat cheese, rice, and bread, with a limited contribution (1 portion/day) of fruits and vegetables, legumes, nuts and seeds, juices, olive oil, coffee, and tea. Participants were provided with several printed low-polyphenol menus; the study nutritionist contacted participants regularly, and 3-day dietary records were collected and reviewed at each visit to monitor adherence. Volunteers completed a questionnaire developed by the Spanish Association for the Study of Menopause (AEEM), which includes items on educational level, relationship status, employment situation, sexual activity, and alcohol and tobacco consumption [6].

Study 2 aimed to (i) assess the distribution of polyphenol metabotypes and MCs and compare them with those of Pre-M women; (ii) evaluate the impact of chronic polyphenol consumption on these distributions; and (iii) explore the effect of PPs on the QoL of Post-M women, as assessed by the C-SF scale, following 8 weeks of PP intake compared to baseline and placebo.

After providing informed consent, Post-M participants consumed three capsules of PPs daily in the evening for three consecutive days. This intake was used to determine the baseline distribution of metabotypes and MCs, analogous to the metabotyping performed in Pre-M participants. Baseline clinical QoL assessments were performed prior to any supplementation, immediately after informed consent was obtained. At that same visit, participants received the capsules for the 3-day metabotyping intake, which was completed before randomization. This distinction ensures that QoL baseline values reflect participants’ unsupplemented status.

**Figure 1:**
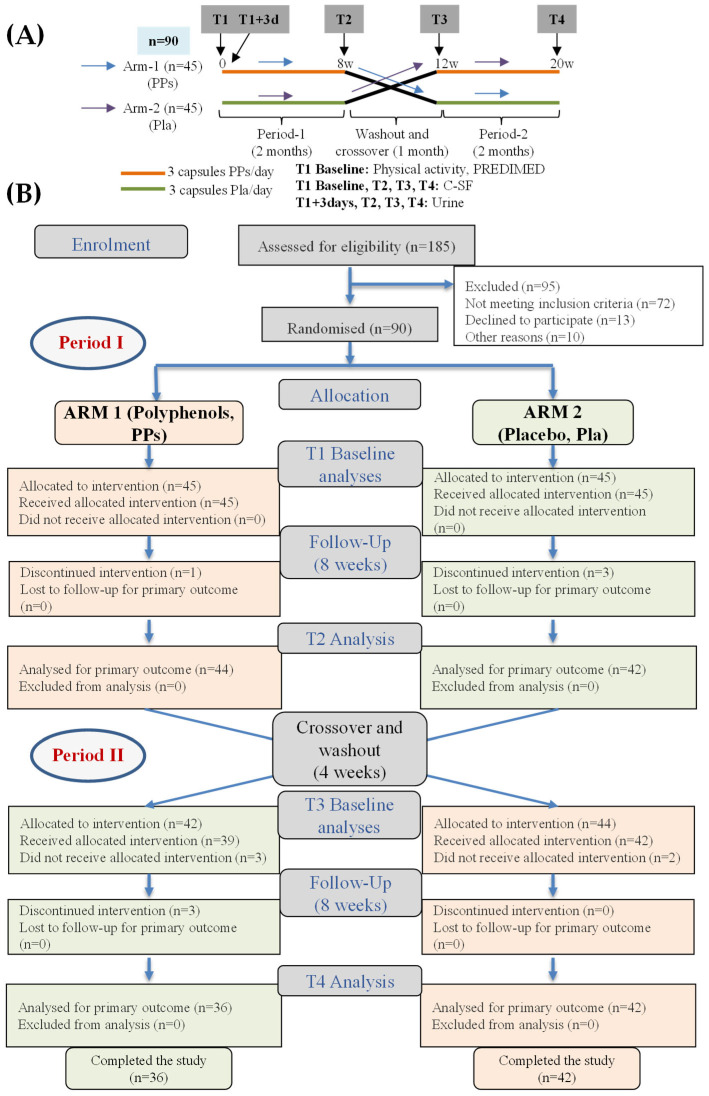
(**A**) Study 2 design. (**B**) Flow chart of Study 2 (CONSORT diagram).

Study 2 employed a crossover design (Figure 1), which allowed for within-subject comparisons across conditions, minimized inter-individual variability, enhanced statistical power, and reduced the sample size required to detect significant effects. Post-M participants were randomized in a 1:1 ratio using computer-generated numbers, with constraints to ensure equal allocation to each treatment sequence. No blocking or stratification was applied. Participants consumed either three capsules of PPs daily in the evening or the placebo (Pla) for 8 weeks. Capsule intake ceased at the end of the first period, followed by a one-month washout, during which participants continued the controlled diet. They then crossed over to the second period, switching treatments so that those who had received PPs now received Pla, and vice versa, again for 8 weeks. Participants were instructed to return any remaining capsules after each study period. Adherence was calculated as the percentage of capsules consumed relative to the expected intake. The total follow-up lasted 5 months. At each sampling point (T), participants provided a urine sample, completed the C-SF, and reported any adverse events or protocol-related incidents (Figure 1).

### 2.4. Cervantes Scale—Short Form (C-SF)

Participants completed the abbreviated version of the Cervantes scale (C-SF) at the four sampling points of the trial (Figure 1A). The C-SF comprises 16 items distributed across four dimensions: menopause and health (with nine subdomains, including vasomotor symptoms, general health, and aging), psychology (three subdomains: anxiety, depression, and fatigue), sexuality (two subdomains: importance of sex and satisfaction with sexual life), and couple relationship (two subdomains: happiness in the relationship and perceived role within it). Each item is scored from 0 (best) to 5 (worst). Higher overall scores indicate greater impact of menopausal symptoms and, consequently, poorer QoL. The collected score was entered into the AEEM calculator, a validated tool that measures postmenopausal QoL from 0 (no impact) to 100 (maximum impact). The C-SF version captures the same health constructs as the larger Cervantes scale but offers greater ease of use in clinical practice [5].

A change of 6.7 points in the global score has been proposed as the minimal clinically important difference (MID) for the C-SF, based on its association with functional outcomes, including sleep quality, productivity, and healthcare utilization [6]. Although no MID thresholds have been formally established for individual domains, proportional estimates based on item distribution suggest that MID may correspond to approximately 3.1 points for the menopause and health domain and 1.6 points for the psychological domain. All these values were used as reference thresholds to interpret the magnitude of domain-specific changes observed in the present study.

### 2.5. Sampling Procedure, Processing, and Analysis

Urine samples were centrifuged at 14,000× *g* for 10 min, filtered through a 0.22 μm PVDF filter, and diluted 1:10 with Mili-Q water containing 0.1% formic acid. Hesperetin (5 μM) was used as an internal standard. It was added to each sample after extraction before analysis. As previously reported [36], diuresis was standardized by measuring urinary creatinine excretion.

Analyses were performed via UPLC-QTOF-MS using an Agilent 1290 Infinity UPLC system coupled to a 6550 accurate-mass quadrupole-time-of-flight (QTOF) mass spectrometer (Agilent Technologies, Waldbronn, Germany) equipped with an electrospray interface (Jet Stream Technology; Agilent Technologies). Separation was carried out using an InfinityLab Poroshell 120 EC-C18 column (3 × 100 mm, 2.7 μm; Agilent Technologies) operating at 30 °C. The mobile phase consisted of water (solvent A) and acetonitrile (solvent B), both of which contained 0.1% formic acid. A flow rate of 0.4 mL/min was applied, and the elution gradient was set as follows: 0–3 min, 5–15% B; 3–11 min, 15–30% B; 11–14 min, 30–50% B; 14–16 min, 50–90% B; 16–18, 90% B; 18–20 min, 95–5% B. Finally, an isocratic step of 1 min duration at 5% solvent B was performed, resulting in a total separation time of 21 min. The ESI operated with the parameters reported elsewhere [36]. Data were processed using Mass Hunter Qualitative Analysis software (version B.08.00, Agilent Technologies, Waldbronn, Germany).

### 2.6. Identification and Quantification of Metabolites

Metabolites were identified by direct comparison with available analytical standards whenever possible. Spectral properties, including molecular mass and fragmentation patterns, were also evaluated to confirm assignments. Tentative identification was performed using the same criteria when standards were unavailable. Additionally, extracted ion chromatograms (EICs) with a narrow mass extraction window (0.01 *m/z*) were used for area calculation and quantification, thereby minimizing the risk of misinterpreting overlapping peaks [36].

All metabolites used to classify participants into the different metabotypes, i.e., equol producers (EP), urolithin A producers (UMA), and IsoUrolithin A and urolithin B producers (UMB), were quantified using validated UPLC-ESI-qTOF-MS methods developed and reported previously under our analytical conditions [36,40]. These validated methods (calibration curves, limits of detection and quantification, repeatability, and recoveries) ensured accurate identification and quantification of phase II metabolites (sulfates and glucuronides) in biological matrices.

For other metabolites lacking commercial standards (e.g., isourolithin A sulfate), LOD and LOQ values could not be experimentally established. These compounds were therefore tentatively identified based on their accurate mass, isotopic pattern distribution, and molecular formula and were included in the analysis only when the signal-to-noise ratio (S/N) exceeded 10, ensuring confident detection and analytical reliability. The identification of lunularin conjugates was performed following the same analytical criteria and methodology previously described by our group [25], which allowed for consistent characterization of lunularin producers and non-producers in human studies.

### 2.7. Statistical Analyses

The impact of PPs intake on C-SF scores was evaluated using a two-way repeated-measures ANOVA model, assessing intra- and inter-group changes in the dependent variable across time and treatment conditions (SigmaPlot v. 16.0, Systat Software, San Jose, CA, USA; the jamovi project 2025, jamovi v. 2.6.26, retrieved from https://www.jamovi.org, accessed on 12 July 2025; Sydney, Australia). This model enabled us to assess the main effects of each factor and potential interactions among them. The Shapiro–Wilk test was applied to examine the normality of the data. When significant differences were detected, post hoc multiple comparisons were performed using the Tukey test to identify specific contrasts between groups.

Analyses were conducted both by considering all participants as a single group and by stratifying them according to individual metabotypes and their clusters (MCs). Changes in C-SF scores were based on least-squares means (LSM) derived from a two-factor repeated-measures Analysis of Variance (ANOVA), which accounted for within-subject variability and the study’s crossover design. Degrees of freedom were adjusted using the Greenhouse–Geisser correction when the assumption of sphericity was violated, as indicated by non-integer residual degrees of freedom. This approach provides adjusted estimates for each condition, enabling robust inferences regarding treatment effects.

To explore the potential influence of covariates, changes in C-SF scores (post-treatment vs. baseline) were correlated with BMI, chronological age, age at menopause onset, years since menopause, and PREDIMED score, using multiple linear regression models or Pearson or Spearman coefficients, depending on data distribution. Comparisons between two independent groups (e.g., Pre-M vs. Post-M at baseline) were performed using the independent *t*-test for normally distributed data or the Mann–Whitney U test for non-normal data. When comparing two dependent groups (e.g., before vs. after PPs or placebo within each arm during both trial periods, i.e., before and after washout and crossover), paired Student’s *t*-tests were used for normally distributed data and the Wilcoxon signed-rank test for non-normal data.

Participants were classified into low, medium, and high producers using unsupervised K-means clustering (k = 3) based on creatinine-normalized metabolite intensities. Data were log-transformed and autoscaled prior to analysis in MetaboAnalyst 5.0 (https://www.metaboanalyst.ca, accessed on 15 September 2025). Clustering was performed using the Hartigan–Wong algorithm with Euclidean distance. The thresholds defining the three clusters correspond to the centroid values automatically determined by the algorithm. For each group, the sum of the specific metabolites was considered to define the production groups as follows: equol producers (EP), including equol glucuronide and sulfate isomers; lunularin producers (LP), including lunularin glucuronide and sulfate isomers; urolithin A producers (UMA), including urolithin A glucuronide and urolithin A sulfate; and urolithin B producers (UMB), including isourolithin A glucuronide and sulfate as well as urolithin B glucuronide and sulfate. For visualization purposes, the clusters were represented using non-log-transformed values to reflect the actual relative metabolite concentrations in each group. Urine samples were collected three days after polyphenol mixture intake in Pre-M women. In Post-M women, samples were collected three days after intake, following the baseline visit (T1 + 3d), and again after eight weeks of supplementation. Data plots were performed using GraphPad Prism 9.1.1 software (GraphPad Software, San Diego, CA, USA) and Sigma Plot 16.0. Statistical significance was set at * *p* < 0.05.

## 3. Results

### 3.1. Characteristics of Participants, Metabotypes, and Metabotype Clusters Distribution at Baseline

Table 2 shows the main participants’ characteristics at baseline. In Pre-M, 160 eligible participants were contacted, and 120 agreed to participate (Study 1). Among Post-M participants, 185 were contacted, and 90 were enrolled in Study 2 (Table 2; Figure 1).

**Figure 2:**
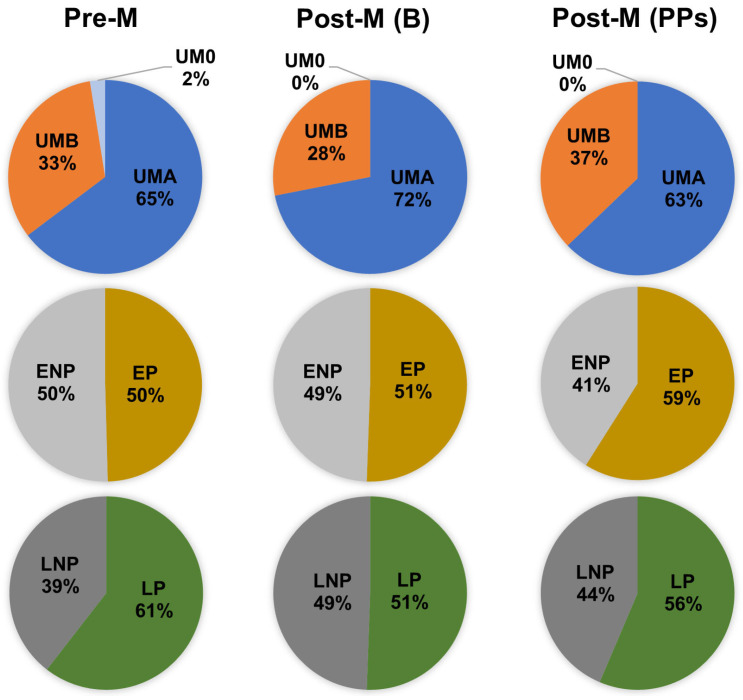
Distribution (%) of individual metabotypes in premenopausal (Pre-M) and postmenopausal women at baseline (Post-M (B)) and after 8 weeks of PP intake (Post-M (PPs)). The baseline was defined as the status after the minimal intake required for metabotype identification (three capsules/day of PPs for 3 days) in both the Pre-M and Post-M (B) groups. Individual metabotypes were also determined in Post-M women after the 8-week PPs intervention (Post-M (PPs)). PPs, a mixture of polyphenol-rich extracts containing resveratrol (RSV), ellagitannins, ellagic acid, and isoflavones (see Materials and Methods). UMA, urolithin metabotype A; UMB, urolithin metabotype B; UM0, urolithin metabotype 0; EP, equol producers; ENP, equol non-producers; LP, lunularin (LUNU) producers; LNP, LUNU non-producers.

The distribution of individual metabotypes was broadly similar between Pre-M and Post-M women at baseline (Table 2; Figure 2). For urolithin metabotypes, UMA was the most prevalent in both groups, with a slight increase from 64.7% in Pre-M women to 71.9% in Post-M women. UMB showed a decrease (32.8% to 28.1%), and UM0 was detected at a low frequency (2.5%) in Pre-M and was absent in Post-M. EP and ENP showed nearly identical distributions across groups (≈50%). Finally, LP decreased from 60.5% in Pre-M to 50.6% in Post-M. Taken together, these findings indicate that menopausal status exerts only a minor influence on the distribution of individual metabotypes in the studied population.

**Figure 3:**
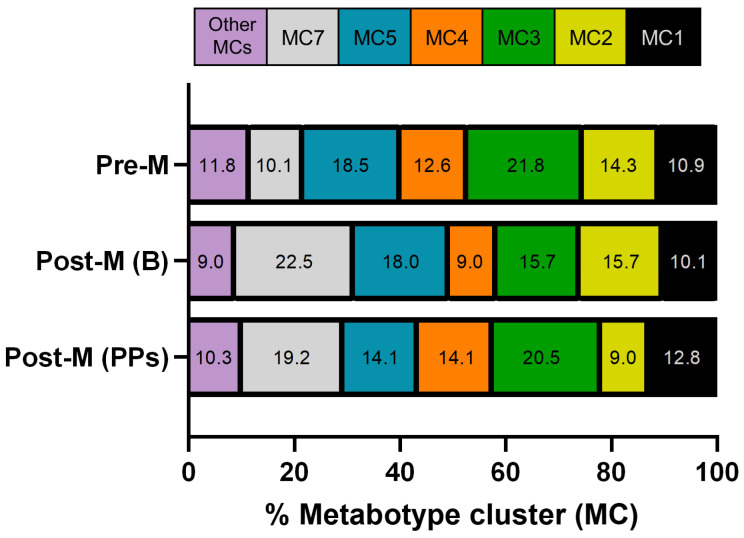
Distribution (%) of metabotype clusters (MCs) in premenopausal (Pre-M) and postmenopausal women at baseline (Post-M (B)) and after 8 weeks of PPs intake (Post-M (PPs)). See Figure 2 and Materials and Methods for details. **MC1**, UMB+ENP+LP; **MC2**, UMA+ENP+LP; **MC3**, UMA+EP+LP; **MC4**, UMB+EP+LP; **MC5**, UMA+ENP+LNP; **MC7**, UMA+EP+LNP; Other MCs: **MC6**, UMB+ENP+LNP; **MC8**, UMB+EP+LNP; **MC9**, UM0+EP+LNP; **MC10**, UM0+ENP+LNP; **MC11**, UM0+EP+LP; **MC12**, UM0+ENP+LP.

The distribution of MCs showed some differences between Pre-M and Post-M women (Table 2; Figure 3). In Pre-M, MC3 was the most prevalent (21.8%), followed by MC5 (18.5%), MC2 (14.3%), and MC4 (12.6%). MC1 and MC7 were less frequent (10.9% and 10.1%, respectively), whereas MC6, MC8, MC9, MC10, and MC11 showed very low prevalence (<6%), and MC12 was not detected. In Post-M, MC7 became the most prevalent cluster (22.5%), followed by MC5 (18.0%) and MC2 and MC3 (15.7% each). MC1 and MC4 had intermediate prevalence (10.1% and 9.0%, respectively), while MC6 and MC8 were infrequent (<6%). Finally, MC9–MC12 were absent.

Overall, MC5 and MC2 were among the more common clusters in both groups. The main difference was a shift in dominance from MC3 in Pre-M to MC7 in Post-M, along with the disappearance of MC9–MC11 after menopause. These findings suggest only moderate changes in MC distribution associated with menopausal status.

### 3.2. Effect of Polyphenol-Rich Extracts Intake on the Distribution of Metabotypes and Metabotype Clusters in Postmenopausal Women

Regarding Study 2 (Figure 1), 78 Post-M participants completed the 5-month follow-up trial. The adherence to the low-polyphenol regimen was high, and 100% of scheduled 3-day dietary records were returned and reviewed. Compliance with the capsule-intake protocol was nearly complete; only a few volunteers returned capsules corresponding to several days, all from the start of the first period. A few volunteers reported some minor side effects after taking the polyphenol-rich mixture (PPs), including mild gastrointestinal discomfort and gas. However, these effects were transient and did not result in any dropouts from the study.

After 8 weeks of PPs intake in Post-M, some shifts in the distribution of individual metabotypes were observed (Figure 2). UMB increased from 28.1% at baseline to 37%, whereas UMA consequently decreased from 71.9% to 63%, and UM0 remained absent. EP increased slightly (from 50.6% to 59%), with a corresponding decline in ENP (from 49.4% to 41%). LP also increased compared with baseline (56% vs. 50.6%), reversing the nearly balanced distribution observed before the intervention. Again, these changes suggest a modest rebalancing of urolithin, equol, and lunularin metabotypes with PPs intake. However, the overall distribution remained broadly similar to both baseline values and those observed in Pre-M women.

Regarding metabotype clusters (MCs), the most prevalent after intervention were MC3 (20.5%) and MC7 (19.2%), followed by MC1 (12.8%), MC4 (14.1%), and MC5 (14.1%) (Figure 3). The remaining, less prevalent clusters, grouped as “Other,” accounted for 10.3%. Therefore, compared with the baseline in Post-M, PPs intake decreased slightly in MC7 (22.5% to 19.2%), whereas MC3 increased (15.7% to 20.5%). MC1 and MC4 both showed higher frequencies than at baseline (10.1% to 12.8% and 9.0% to 14.1%, respectively), while MC2 declined (from 15.7% to 9.0%) (Figure 3). In comparison with the Pre-M group, the distribution of MCs in Post-M after consuming PPs still differed in the relative prominence of MC7 over MC3. However, the gap narrowed due to the increase in MC3 with PPs intake (Table 2, Figure 3). Overall, PPs intake led to moderate shifts in individual metabotypes and MCs in Post-M women, with partial convergence toward Pre-M patterns, but no significant reconfiguration of metabotypes and MCs prevalence.

### 3.3. Quantitative Production of Metabolites in Premenopausal and Postmenopausal Women at Baseline

Following the intake of 3 capsules of PPs per day for three consecutive days, no significant differences (*p* > 0.05) were observed using the *t*-test or Mann–Whitney–Wilcoxon tests in the quantitative production of metabolites derived from Uro-A (produced in UMA), Uro-B and IsoUro-A (UMB), equol (EP), and LUNU (LP) between Pre-M and Post-M women at baseline. Production of Uro-A-derived metabolites was 1.38-fold higher in Pre-M compared to Post-M (*p* = 0.417). For Uro-B-derived metabolites, production was 1.15-fold higher in Post-M versus Pre-M (*p* = 0.656). Equol production was 1.2-fold higher in Pre-M women (*p* = 0.216), and lunularin-derived metabolites were 1.3-fold higher in Pre-M versus Post-M (*p* = 0.688). Therefore, menopausal status per se did not significantly affect the quantitative production of these metabolites.

### 3.4. Distribution of Low-, Medium-, and High-Metabolite Producers in Premenopausal and Postmenopausal Women at Baseline

**Figure 4:**
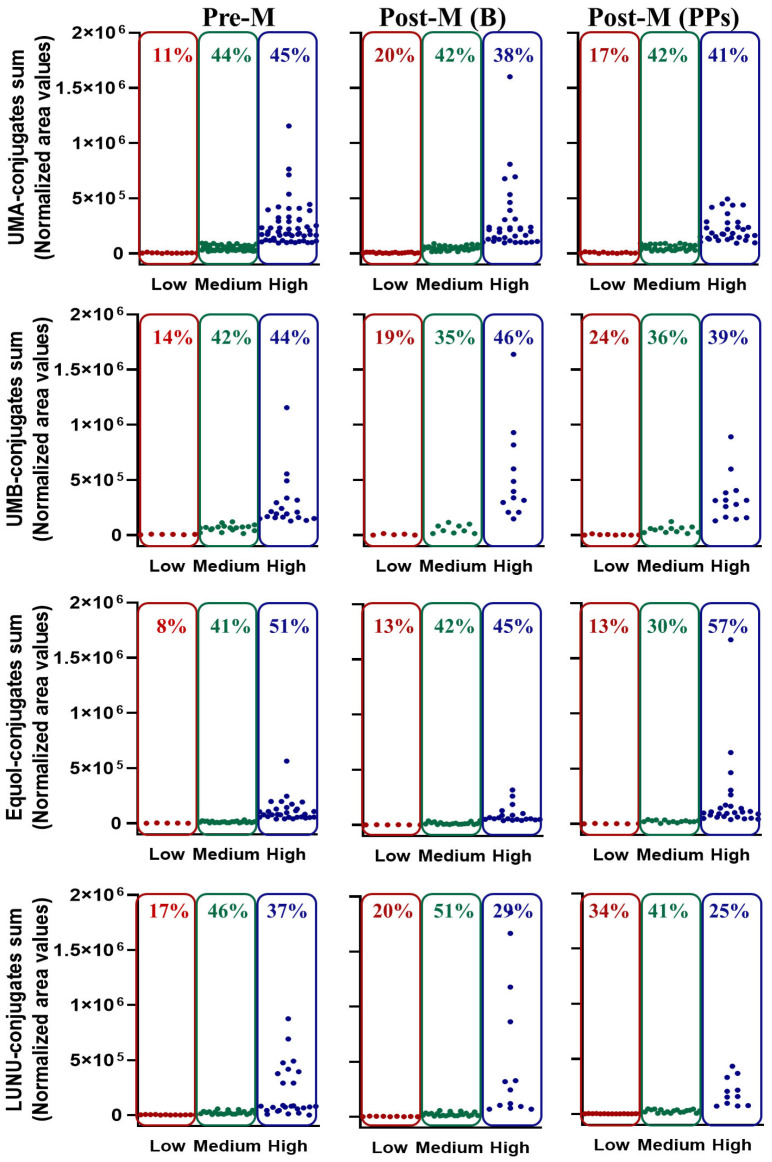
Clustering of volunteers by high-, medium-, and low-metabolite conjugates (Uro-A, Uro-B+IsoUro-A, equol, and LUNU) excretion in urine. Pre-M, premenopausal women; Post-M (B), postmenopausal women at baseline; Post-M (PPs), postmenopausal women after consuming PPs for 8 weeks. Red, low; green, medium; blue, high. Metabolite values (Extracted Ion Chromatogram peak intensity) were standardized by urine creatinine concentration. UMA, Uro-A conjugates in metabotype A; UMB, Uro-B and IsoUro-A conjugates in metabotype B; LUNU, lunularin conjugates in LP.

Next, we examined the distribution of low, medium, and high producers for these metabolites between Pre-M and Post-M women at baseline (Figure 4). For this analysis, representative conjugated metabolites from each metabotype were included: for UMA, Uro-A glucuronide and Uro-A sulfate; for UMB, IsoUro-A glucuronide, IsoUro-A sulfate, Uro-B glucuronide, and Uro-B sulfate; for EP, equol glucuronide and equol sulfate isomers; and for LP, LUNU glucuronide and LUNU sulfate isomers. A higher percentage of low producers was observed in the Post-M group: 5% more for metabolites derived from UMB and equol, 10% more for UMA derivatives, and 3% more for LP. A lower percentage of high producers was also observed for each metabolite class, with a particularly notable 8% decrease in LP high producers in Post-M compared to Pre-M.

### 3.5. Effect of Chronic PPs Intake on Metabolite Production in Postmenopausal Women

No significant differences in metabolite production were observed in Post-M women after PP consumption for Uro-A, Uro-B, and lunularin metabolites when analyzed using the Mann–Whitney U test. Production of Uro-A-derived metabolites was 1.3-fold higher after PP intake (*p* = 0.966). In the case of Uro-B-derived metabolites, production was 1.6-fold lower after PP consumption (*p* = 0.768). Finally, production of lunularin-derived metabolites was 1.1-fold lower following PP intake in Post-M women (*p* = 0.441). However, in EP, the median equol production increased 2.8-fold (mean of 13.6 ± 5.5 SEM) following PPs intake, with 86% of EP showing an increase in equol concentration by the end of the intervention.

### 3.6. Distribution of Low, Medium, and High Producers Following PPs Intake in Postmenopausal Women

Regarding the distribution of low, medium, and high producers of metabolites after 8 weeks of PPs intake in Post-M women, no apparent shift was observed, contrary to expectations, from low to higher producers (Figure 4). In contrast, the proportion of low producers remained stable or even increased for LUNU derivatives. Only in EP was a trend toward increased high producers observed, while the percentage of low producers remained unchanged.

### 3.7. Impact of Chronic PPs Intake on the Quality of Life of Postmenopausal Women

PPs supplementation over 8 weeks resulted in statistically significant improvements in QoL scores on the C-SF compared with placebo (Pla), both in the global score and the menopause and health domain. In the full sample (*n* = 78), the effects of PPs intake were statistically significant but not clinically relevant (score reduction of −6.7 or lower) compared with placebo for both the global (*p* = 0.023) and menopause and health domains (*p* < 0.001) (Table 3). Within the PPs group, all domains showed significant (*p* < 0.05) pre–post improvements, except for sexuality and couple relationship (Table 3). More importantly, clinically relevant changes (equal to or above the MID, i.e., score reduction ≥ −6.7 points) were observed in specific metabotypes and MCs, such as EP, UMB, MC3, and MC4, with improvements ranging from 20% (UMB) to 41.3% (MC4) in the Menopause and Health domain. Although the clinically meaningful change for a subdomain such as hot flashes has not been established, the robust and significant reduction (−10.4 LSM, PPs vs. baseline; *p* = 0.001), together with the 95% CI not crossing zero, and a considerable effect size (Cohen’s d = −3.39 for PPs vs. placebo), strongly suggests that the improvement observed in the Health domain was mainly driven by the marked decrease in hot flashes incidence among EP volunteers. In the Psychic domain, a clinically relevant change was detected when considering all participants, specifically in the EP metabotype and MC3, both of which are equol producers (Table 3). Thus, although this relevant effect was observed in the overall group, it was primarily driven by participants who produce equol. These results suggest that the impact of PPs was statistically detectable and, more importantly, clinically meaningful, particularly in the EP metabotype within the domains of menopause and health and psychic well-being. Specifically, in EP participants (*n* = 46), 78% showed a reduction in the Menopause and Health domain score after PPs intake, and 63% achieved a clinically meaningful improvement (≥6.7-point reduction), with a 95% confidence interval of 48.7–75.7%. In the Psychic domain, 71% of EP participants improved, and 58% reached the MID threshold, with a 95% confidence interval of 43.3–71.6%.

**Figure 5:**
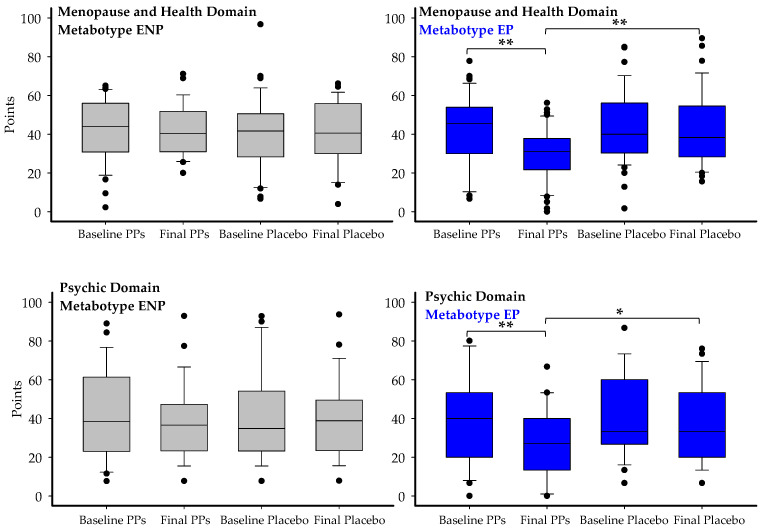
Changes in Cervantes scale-SF scores in the Menopause and Health domain and the Psychic domain, comparing ENP and EP metabotypes. Box plots represent baseline and post-intervention scores for polyphenols (PPs) and placebo in each domain and metabotype. In the EP group, a significant improvement is observed after PPs intake (*p* < 0.05), which is not maintained during the subsequent placebo phase, as scores return to baseline levels, suggesting the absence of carry-over effects. No significant changes are observed in the ENP group. Asterisks indicate statistically significant differences: * *p* < 0.05, ** *p* < 0.01.

The participants’ diet did not influence the observed effects, as adherence to the low-polyphenol diet was high, and all reported a strong adherence to the Mediterranean diet in their usual dietary habits. Multiple linear regression models were used to assess the impact of diet while controlling for BMI, age, and ethnicity (98% Caucasians), confirming that these covariates did not significantly affect the outcomes. It is essential to highlight that no carry-over effect was observed. Figure 5 illustrates differences observed in the Menopause and Health and Psychic domains, comparing ENP and EP metabotypes. After PP intake, scores do not remain low but return to baseline levels, indicating no carry-over effect. A defined response is observed in EP but not in ENP, and no significant difference is seen between PPs and placebo.

## 4. Discussion

The different responses of individuals to polyphenol consumption have also been observed in studies on polyphenols and quality of life (QoL) in postmenopausal (Post-M) women. Indeed, evidence linking polyphenol intake to improvements in Post-M QoL is inconsistent. Some studies report benefits from resveratrol (RSV) [41,42], RSV plus equol [43], 8-prenylnaringenin, isoflavones, and melatonin [39], or pomegranate and isoflavones [44]. In contrast, cocoa-rich chocolate [45] or blackcurrant polyphenols [46] showed no effect on QoL. In addition, a recent meta-analysis of ten randomized controlled trials involving 928 participants concluded that RSV did not significantly impact QoL [19].

Although the different outcomes of these studies may be due to multiple factors (participants’ genetics, ethnicity, clinical trial protocols, lack of control over key factors such as medication, etc.), one aspect that may contribute is the differential metabolism of polyphenols among individuals, that is, according to their polyphenol-related gut microbiota metabotype [9,11,21].

In this regard, we describe here, for the first time, the polyphenol metabotyping and MC clustering of postmenopausal (Post-M) women. The overall distribution of polyphenol metabotypes appeared relatively stable across menopausal status, supporting the idea that, although estrogen decline impacts the gut microbiota composition [47], other factors, such as aging, obesity, or chronic medication, are more decisive in shaping these polyphenol metabotypes [9,23,31,32,33].

In the present study, the percentage of UMA and UMB in Pre-M women roughly matched that described in previous studies on healthy adults [24,31], with UMA increasing to 72% in Post-M women after chronic intake of PPs. Remarkably, urolithin metabotyping is typically performed after three days of consuming ellagitannin-rich sources [24]. Under that protocol, the abundance of metabotype 0 (UM0) in healthy individuals has been reported at approximately 10% [24,31], with variations depending on geographic location, population size, and analytical sensitivity [48,49].

However, we postulate that when the duration of polyphenol intake increases, the likelihood of detecting UM0 decreases. For example, Iglesias-Aguirre et al. [22] reported 1.5% UM0 in 127 healthy adult men and women after one week of consuming a pomegranate extract rich in ellagic acid and other polyphenols. In the present study, 2% of Pre-M women and 0% of Post-M women were classified as UM0 at baseline. Overall, the percentage of UM0 individuals in this population appears to be lower than previously reported [24,31]. It has been suggested that the amount of metabolite produced, in addition to the minimal abundance of gut bacteria involved in its synthesis, may be strongly influenced by external variables, such as the dose of precursor polyphenol, gastric motility, the time between intake and detection [11], and also the duration of intake. For this reason, the percentage of UM0 in Pre-M women might have been even lower if they had consumed PPs for a longer period, rather than just 3 days. Overall, our results indicate that prolonged intake of high concentrations of ellagic acid and ellagitannins may convert apparent non-producers of urolithins (UM0) into producers of metabotype A (UMA) or B (UMB), as previously observed [26]. However, this increase in production might occur only in specific individuals with low relative abundance of urolithin-producing bacteria, because overall, and perhaps surprisingly, continued intake over eight weeks of high concentrations of ellagic acid and ellagitannins did not increase urolithin production in Post-M women. Overall, this suggests an apparent saturation in metabolite production after eight weeks of PPs intake, observed for both urolithins and LUNU derivatives. However, we noticed a clear increase in equol production among EP Post-M women. Since no mechanistic data were collected, possible explanations, such as low baseline abundance of urolithin-producing taxa, microbial resilience, substrate competition, or feedback inhibition, are speculative and require targeted mechanistic studies to confirm.

Regarding EP, it is also worth noting that the baseline percentage found in both Pre-M and Post-M women (≈50%) was higher than that reported in other studies for Western populations (≈30%) [23]. This increase is also attributed to the fact that our cohort consisted of women, as equol production has been reported to be higher in women than in men [49]. In our study, the prevalence was similar to that reported in Japanese female populations (47%) [50], increasing to 59% in Post-M women after chronic PPs intake. Finally, all Pre-M and Post-M produced the metabolite O-demethylangolesin (ODMA). Although the existence of an ODMA-related metabotype has been suggested [51], our results do not confirm this metabotype, in agreement with [22], who also observed the production of this metabolite in all adult male and female participants (*n* = 127). Since ODMA can be further metabolized into simpler metabolites, its detection may be influenced not only by gut microbiota composition but also by other variables involved in the metabolism and clearance of these compounds, such as intestinal motility, the time from intake to excretion, and the sensitivity of analytical methods [9,11]. Thus, when a metabolite is not detected, it may reflect either a true lack of production or a failure to capture it due to these factors. Conversely, when it is unequivocally detected, there is no doubt about the individual’s ability to produce it.

Regarding RSV metabolism, no previous studies have assessed the effect of chronic RSV intake on the percentage of LP and LNP metabotypes. In healthy young adults, approximately 75% were LP and 25% LNP after seven days of RSV consumption [22,25]. In our study, we observed a slightly lower proportion in Pre-M women (61% LP) and an even lower percentage in Post-M women at baseline (51%), which increased to 56% after eight weeks of intake. This lower percentage of LP aligns with the previously suggested trend, indicating lower LUNU production in females compared to males [25]. Overall, chronic PPs intake in Post-M women seemed to shift individual metabotype percentages toward those observed in Pre-M women.

Regarding MCs, only one previous study has reported their distribution in young adults [22], where MC1 and MC2 were most prevalent. In the present research, MC3 and MC5 were most abundant in Pre-M women, mainly due to the higher percentage of EP individuals in this female-only cohort. In Post-M women, MC5 and MC7 were most abundant at baseline, but following PPs intake and the resulting increase in EP, the most prevalent clusters shifted to MC7 and MC3.

Overall, this entire puzzle of metabotypes and their combinations (MCs) may be relevant to the health impact of ingested polyphenol precursors. Indeed, our study highlights their relevance as key modulators of the effects of chronic supplementation of RSV, ellagitannins, and isoflavones on QoL in Post-M women. In this regard, the intervention revealed that the benefits of PPs supplementation were not uniformly distributed across individuals but depended on specific metabotypes and MCs. Clinically meaningful improvements in QoL, particularly in the Menopause and Health and Psychic domains, were observed primarily among equol producers (EP), where the requirements for statistical significance and clinical relevance were met, given the necessary sample size. In the case of other subgroups (i.e., MC3 and MC4), although a large effect was detected (a much smaller drop of −6.7 points), the sample size suggests that statistical power may be insufficient. Therefore, in these cases, these effects should be considered exploratory until validated with larger sample sizes. Overall, these results highlight the role of microbial-derived metabolites in modulating intervention outcomes. Although the involvement of other compounds present in the tested extracts and/or the precursor polyphenols cannot be ruled out, our results support that equol is a major contributor, consistent with its reported pleiotropic health effects [52,53,54,55,56]. It is also important to emphasize the difference between statistical significance and clinical relevance; for example, for the impact on the global domain in the whole group (Table 3): although it is statistically significant (end vs. baseline and vs. placebo), the change (−3.4) after PPs consumption does not reach the MID threshold (−6.7).

Consequently, by considering metabotype variability, our results suggest a resolution, at least partially, to the apparent inconsistency between polyphenol intake and health outcomes. This means that the positive effects of polyphenols may not be generalizable to the entire population but may instead be concentrated in subgroups defined by their metabolic potential for certain health indications [11,21].

Although broader generalization is limited by sample size, these findings provide a solid foundation for future research on metabotype-guided strategies in personalized nutrition and women’s health [9]. Identifying individuals based on their ability to produce specific microbial-derived metabolites may optimize dietary interventions and prevent dilution of effects when results are analyzed in heterogeneous populations. The use of MCs provides an additional conceptual framework, as it captures the combined influence of multiple metabotypes rather than focusing on a single metabolite pathway [9,22].

Some limitations should be acknowledged. The study was not designed to track mechanistic changes in gut microbiota since this approach is ongoing. Additionally, although the design was placebo-controlled and crossover to address interindividual variability, it should be noted that subgroup analyses (i.e., analyses considering specific metabotypes or MCs) inevitably reduce statistical power. Although the number of participants who completed the study exceeded the planned sample for the secondary QoL outcome (*n* = 78 vs. *n* = 40), findings from post hoc analyses in subgroups with *n* < 40 should be considered exploratory and require replication in adequately powered trials. Consequently, analyses in subgroups with *n* ≥ 40 retain greater statistical power and are not labeled exploratory by default. Future research should integrate metagenomics and metabolomics to clarify the microbial determinants of metabotypes and confirm the durability of the observed clinical benefits.

In summary, our findings underscore that metabotypes and MCs modulate the impact of polyphenol supplementation on the QoL of Post-M women. This work supports the development of targeted dietary strategies, transitioning from a “one-size-fits-all” approach to interventions informed by gut microbial metabolism and interindividual variability [21,57]. Our ongoing research into the effects of menopausal status and chronic polyphenol intake on gut microbiota and health outcomes seeks to support precision health strategies for women in postmenopause, a pivotal stage in their lives.

## 5. Conclusions

This study is the first to describe the distribution of polyphenol-related metabotypes and their combinations (MCs) in postmenopausal (Post-M) women. The intervention showed that improvements in QoL, particularly in menopause-related and psychological domains, were not uniformly distributed but concentrated among equol producers (EP) and specific MCs (MC3 and MC4). These results indicate that the clinical impact of a mixture of polyphenol-rich plant extracts (RSV, pomegranate, and red clover) on QoL depends on the individual’s metabolic capacity to generate specific bioactive microbial-derived metabolites, in this case, driven mainly by equol production.

By identifying these metabotype-dependent effects, the study provides a mechanistic explanation for previously inconsistent outcomes and supports the use of metabotyping and MC profiling to guide targeted dietary strategies. This approach may increase the efficacy of interventions and advance precision health in Post-M women.

## Figures and Tables

**Table 1:** Composition of phenolic compounds in the plant extract mixture (PPs) ^1^.

Compound	RT	*m/z^-^*	MS/MS	λ_max_	mg/g Extract	mg/Capsule
**Pomegranate**						
Punicalin	12.76	781	721/601/299	258/380	11.47 ± 0.92	8.03 ± 0.65
Punicalagin α	15.08	1083	781/721/601	258/372	48.15 ± 8.75	33.71 ± 6.13
Punicalagin β	17.08	1083	781/721/601	258/372	48.20 ± 6.24	32.16 ± 2.28
Ellagic acid	25.68	301	257/229/185	254/360	42.81 ± 1.81	29.97 ± 1.27
**Σ Ellagitannins and ellagic acid**						**103.9 ± 10.32**
**Resveratrol**						
*trans*-Resveratrol	30.84	227	185/157	306	63.42 ± 4.80	44.39 ± 3.36
**Red clover**						
Daidzein	32.33	253	225/209	248/304	5.20 ± 0.61	3.64 ± 0.43
Genistein	37.15	269	241/225	260/324	4.86 ± 0.15	3.41 ± 0.10
Formononetin	40.80	267	252/235/211	250/304	43.81 ± 1.60	30.67 ± 1.12
Biochanin A	43.15	283	268/251/227	260/332	25.34 ± 10.63	17.74 ± 7.44
**Σ Isoflavones**						**55.45 ± 9.10**
**Total phenolics**					**203.7 ± 22.8**	

^1^ The identification of compounds was based on their elution order, UV spectra, *m/z*, and MS/MS fragmentation patterns, as well as comparison with authentic standards when available. *trans*-Resveratrol (quantified at 310 nm), ellagic acid (360 nm), punicalagin α and β (360 nm), daidzein (270 nm), genistein (270 nm), formononetin (270 nm), and biochanin A (270 nm) were identified by direct comparison with authentic standards. Punicalin was quantified as the sum of its α and β isomers at 360 nm. Values are expressed as mean ± SD (*n* = 5). RT, retention time.

**Table 2:** Characteristics of premenopausal (Pre-M) and postmenopausal (Post-M) women at baseline.

Characteristics	Values ^1^	
	Pre-M (*n* = 120)	Post-M (*n* = 90)
**Age** (years, y)	40.2 ± 4.0; 41.0 (30−48)	53.2 ± 3.3; 54.0 (45−59)
**BMI** (kg/m^2^)	23.7 ± 3.8; 23.0 (18.3−36.2)	26.0 ± 4.3; 25.1 (19.0−37.4)
Normal weight	85 (70.8%)	45 (50.0%)
Overweight	27 (22.5%)	32 (35.6%)
Obese	8 (6.7%)	13 (14.4%)
**Menopause onset** (y)	-	49.7 ± 3.7 (40−56)
**Postmenopausal duration** (y)	-	3.9 ± 3.5 (0.3−14)
**Domains of the Cervantes Scale:**		
Global		36.7 ± 16.3; 34.1 (5.6−77.2)
Menopause and health	-	45.0 ± 19.3; 42.8 (2.2−96.7)
Psychic		36.7 ± 27.7; 33.3 (0−100)
Sexuality		44.6 ± 24.6; 40 (0−100)
Couple relationship		14.7 ± 20.10; 10 (0−100)
**Individual metabotypes:**		
UMA, UMB, UM0	(64.7%, 32.8%, 2.5%)	(71.9%, 28.1%, 0%)
EP, ENP	(49.6%, 50.4%)	(50.6%, 49.4%)
LP, LNP	(60.5%, 39.5%)	(50.6%, 49.4%)
**Metabotype clusters (MCs):**		
**MC1:** UMB+ENP+LP	10.9%	10.1%
**MC2:** UMA+ENP+LP	14.3%	15.7%
**MC3:** UMA+EP+LP	21.8%	15.7%
**MC4:** UMB+EP+LP	12.6%	9.0%
**MC5:** UMA+ENP+LNP	18.5%	18.0%
**MC6:** UMB+ENP+LNP	6.0%	5.6%
**MC7:** UMA+EP+LNP	10.1%	22.5%
**MC8:** UMB+EP+LNP	3.4%	3.4%
**MC9:** UM0+EP+LNP	0.8%	0%
**MC10:** UM0+EP+LNP	0.8%	0%
**MC11:** UM0+EP+LP	0.8%	0%
**MC12:** UM0+ENP+LP	0%	0%

^1^ Values are shown as mean ± SD, median, and (range), or percentage. The MC numbering follows that previously described, based on the percentage found [22].

**Table 3:** Change and percentage improvement in Cervantes scale-SF scores in postmenopausal women after 8 weeks of PPs intake vs. placebo, adjusted for interindividual variability in a crossover design ^1^.

Domains and Subdomains	Placebo (Pla)	Polyphenols (PPs)	*p*-Value (PPs vs. Pla)	95% CI (LSM) (PPs-Pla)	Cohen’s d
**Global**					
***All*** *(n = 78)*	0.49 ± 1.4; −1.5%	−3.4 ± 1.5; 9.5% *	**0.023**	[−7.9, 0.21]	−0.30
***Metabotypes***					
UMB (*n* = 29)	−1.1 ± 2.3; 3%	−5.1 ± 2.1; 12.8% *	**0.037**	[−7.1, −0.9]	−0.41
EP (*n* = 46)	−2.1 ± 2.2; 5.2%	−6.2 ± 2.1; 16.6% *	**0.039**	[−7.2, −1.2]	−0.28
***MCs***					
MC4: UMB+EP+LP (*n* = 11)	−1.6 ± 1.9; 5%	−6.1 ± 1.8; 15.3% *	**0.048**	[−8.8, −0.3]	−0.58
**Menopause and health**					
***All*** *(n = 78)*	−2.1 ± 2.8; 3.5%	−6.3 ± 1.9; 14.2% *	**<0.001**	[−8.9, 0.5]	−0.20
***Metabotypes***					
UMA (*n* = 49)	−2.6 ± 2.4; 5.5%	−4.7 ± 2.1; 10.5% *	**0.012**	[−5.8, −0.6]	−0.38
UMB (*n* = 29)	−3.1 ± 3.5; 6.2%	−9.3 ± 3.1; 20% *^&^	**0.006**	[−10.3, −2.1]	−0.52
EP (*n* = 46)	−4.3 ± 2.6; 5.2%	−10.1 ± 2.3; 24.1% *^&^	**0.004**	[−9.9, 4.7]	−0.35
***MCs***					
MC4: UMB+EP+LP (*n* = 11)	−4.6 ± 5.1; 8.8%	−20.8 ± 4.3; 41.3% *^&^	**0.022**	[−24, −11.6]	−0.84
MC3: UMA+EP+LP (*n* = 16)	−1.6 ± 3.4; 3.4%	−9.7 ± 4.1; 22.5% *^&^	**0.043**	[−12.6, 2.8]	−0.61
**Psychic**					
***All*** *(n = 78)*	−3.8 ± 2.4; 9.8%	−8.3 ± 2.2; 23.8% *^&^	0.053	[−7.9, −1.7]	−0.41
***Metabotypes***					
EP (*n* = 46)	−3.4 ± 3.1; 8.5%	−9.9 ± 2.9; 28% *^&^	**0.039**	[−8.8, −3.4]	−0.32
***MCs***					
MC3: UMA+EP+LP (*n* = 16)	0.48 ± 5.2; 1.3%	−6.7 ± 4.1; 17.8% *	0.051	[−11.3, −0.1]	−0.61
**Sexuality**	2.7 ± 2.6; −7.5%	−5.1 ± 4.3; 11%	0.309	[−12.3, 1.5]	−0.26
**Couple relationship**	−1.6 ± 2.7; 5.5%	−1.9 ± 2.4; 9%	0.653	[−3.8, 2.2]	−0.06

^1^ Values are shown as Least Squares Means (LSM) ± standard error of the mean (SEM). More negative score values indicate greater improvement. Improvement was calculated as (Initial score–Final score)/Initial score × 100. *p*-values refer to treatment effects (PPs vs. placebo) from a two-factor (time × treatment) repeated measures ANOVA, accounting for interindividual variability and the washout period. Significant differences between placebo (Pla) and PPs are shown in bold. Asterisks (*) indicate significant differences (*p* < 0.05) within treatment (final vs. baseline). The ampersand symbol (^&^) indicates a clinically relevant change, i.e., a value equal to or exceeding the minimal clinically important difference (MID) in the C-SF. Individual metabotypes and/or metabotype clusters (MCs) with no significant differences are not shown. Standardized effect sizes (Cohen’s d) were calculated as the difference between group means divided by a pooled standard deviation derived from SEMs and sample size. Negative values indicate greater improvement in the PPs group vs. placebo. Effect sizes were interpreted using standard thresholds: small (d ≈ 0.2), moderate (d ≈ 0.5), and large (d ≥ 0.8), though clinical relevance may depend on context.

## Data Availability

The datasets generated and analyzed during the current study are not publicly available due to ethical restrictions related to participant confidentiality. However, anonymized individual-level data supporting the main findings are available from the corresponding author upon reasonable request.

## Abbreviations

The following abbreviations are used in this manuscript:

BMI	body mass index
C-SF	Cervantes Scale short form
EA	ellagic acid
EIC	extracted ion chromatogram
EP	equol producer
ENP	equol non-producer
HPLC-ESI-IT-MS/MS	High-Performance Liquid Chromatography coupled to Electrospray Ionization-Ion Trap Tandem Mass Spectrometry
LUNU	lunularin
LP	lunularin producer
LNP	lunularin non-producer
MC	metabotype cluster
MS	mass spectrometer
ODMA	*O*-desmethylangolensin
Pla	placebo
QoL	quality of life
RSV	*trans*-resveratrol
PPs	polyphenol-rich plant extract mixture
Pre-M	premenopausal
Post-M	postmenopausal
SD	standard deviation
SEM	standard error of the mean
UPLC-ESI-QTOF-MS	ultra-high performance liquid chromatography coupled with electrospray ionization and quadrupole time-of-flight mass spectrometry
Uro	urolithin
UMA	metabotype A
UMB	metabotype B
UM0	metabotype 0
